# Immunohistochemistry and real-time Polymerase Chain Reaction: importance in the diagnosis of intestinal tuberculosis in a Peruvian population

**DOI:** 10.1186/s12876-024-03235-6

**Published:** 2024-05-16

**Authors:** Fernando Arevalo, Soledad Rayme, Rocío Ramírez, Romy Rolando, Jaime Fustamante, Mario Monteghirfo, Rocio Chavez, Eduardo Monge

**Affiliations:** 1 Gastroenterology Department, Hospital Nacional Daniel A., Carrión, Lima, Perú; 2 Pathology Department, Hospital Nacional Daniel A. Carrión, Callao, Lima Perú; 3 Instituto de Medicina Legal y Ciencias Forenses – Perú, Lima, Perú; 4 Histodiagnóstico Gastrointestinal Private Pathology Center, Lima, Perú; 5 Universidad Nacional Mayor de San Marcos, Lima, Perú; 6 Departamento de Ciencias Dinámicas, Facultad de Medicina, Instituto de Investigacion de Bioquímica y Nutrición Alberto Guzmán Barrón, Universidad Nacional Mayor de San Marcos, Lima, Perú; 7 Gastroenterology Department, Hospital Nacional Adolfo Guevara Velasco EsSalud, Cuzco, Perú; 8 Universidad San Antonio Abad, Cuzco, Perú; 9 Instituto de Gastroenterologia del Sur, Cuzco, Perú

**Keywords:** Intestinal tuberculosis, Immunohistochemistry, Real-time Polymerase Chain Reaction, *Mycobacterium tuberculosis*, Diagnosis, Histologic findings, Endoscopic findings, Colonoscopy

## Abstract

**Introduction:**

The diagnosis of intestinal tuberculosis is challenging even nowadays. This study aims to report the positivity rates of new diagnostic methods such as immunohistochemistry and Real-Time Polymerase Chain Reaction in patients with intestinal tuberculosis, as well as describe the pathological and endoscopic features of intestinal tuberculosis in our population.

**Methods:**

This was a retrospective observational study conducted in patients diagnosed with intestinal tuberculosis, between 2010 to 2023 from the Hospital Nacional Daniel Alcides Carrion and a Private Pathology Center, both located in Peru. Clinical data was obtained, histologic features were independently re-evaluated by three pathologists; and immunohistochemistry and real-time Polymerase Chain Reaction evaluation were performed. The 33 patients with intestinal tuberculosis who fulfilled the inclusion criteria were recruited.

**Results:**

Immunohistochemistry was positive in 90.9% of cases, while real-time Polymerase Chain Reaction was positive in 38.7%. The ileocecal region was the most affected area (33.3%), and the most frequent endoscopic appearance was an ulcer (63.6%). Most of the granulomas were composed solely of epithelioid histiocytes (75.8%). Crypt architectural disarray was the second most frequent histologic finding (78.8%) after granulomas, but most of them were mild.

**Conclusion:**

Since immunohistochemistry does not require an intact cell wall, it demonstrates higher sensitivity compared to Ziehl–Neelsen staining. Therefore, it could be helpful for the diagnosis of paucibacillary tuberculosis.

**Supplementary Information:**

The online version contains supplementary material available at 10.1186/s12876-024-03235-6.

## Introduction

Tuberculosis is a common disease with a rising incidence in recent years, particularly in developing countries such as Perú [1]. It can affect the gastrointestinal system eventually. In fact, intestinal tuberculosis (intestinal TB) ranks as the sixth most common site of extra-pulmonary involvement and it is the prevailing form of abdominal tuberculosis reported in tertiary care centers [2, 3]. Unfortunately, diagnosing intestinal tuberculosis is quite difficult due to its similarity to other diseases like colon carcinoma or Crohn’s disease. The diagnosis can be delayed because the microbiologic culture results often take 4 to 6 weeks to obtain [4]. Nonetheless, despite that, currently culture remains as the gold standard [5, 6]. The paucibacillary nature of *Mycobacterium tuberculosis* (MT) hinders its detection, which increases the risk of false negatives.

These diagnostics difficulties could lead to empiric treatments [7], patient hospitalization, prolonged illness, and finally death [8]. Consequently, novel diagnostic methods have come out that may help in the diagnosis of intestinal TB like immunohistochemistry or real-time Polymerase Chain Reaction (real-time PCR).

The histological diagnosis of intestinal TB is based on the identification of granulomas and acid-fast bacilli stain positive in the biopsy (Ziehl–Neelsen positive). However, the sensitivity of this test is low and operator-dependent. Furthermore, the granulomas can also be found in other pathologies like Crohn's disease or systemic mycosis [9].

Real-time PCR could be used in tuberculosis diagnosis, and has a higher sensitivity and specificity than culture or stains [10]. IS6110 is a DNA target sequence present only in mycobacteria, that is commonly used for *Mycobacterium tuberculosis*complex detection by real-time PCR [11]. Real-time PCR amplifies and detects specific DNA sequences via fluorescent dyes, which are linked to oligonucleotide probes that bind specifically to the amplified product. The identification and quantification of the accumulating product is achieved by rating the fluorescence intensities during the real-time PCR procedure [12]. However, real-time PCR is still expensive for low-income populations who are precisely the most affected by tuberculosis.

Immunohistochemical study for MT in biopsies is an alternative diagnostic tool that can be considered. This technique is based on the detection of MT antigens in paraffin blocks through polyclonal or monoclonal antibodies [13]. The sensitivity of this test can be extremely high, but the specificity is quite variable according to different authors. On the other hand, compared to real-time PCR, immunohistochemistry is lower cost and easier to perform [14].

The aim of this study is to report the positivity rates of immunohistochemistry and real-time PCR for MT in patients with intestinal TB and describe the pathological features of this disease in our population.

## Methods

This was a retrospective observational study which was conducted at the Hospital Nacional Daniel Alcides Carrion (HNDAC) and Histodiagnóstico Gastrointestinal Private Pathology Center, both located in Lima-Peru; from 2010 to 2023. From which medical records from patients with a diagnosis of intestinal TB (small bowel, colon or rectum) were selected.

We included patients with the diagnosis of intestinal tuberculosis who underwent upper endoscopy or colonoscopy with biopsy, whose diagnosis of intestinal tuberculosis met at least one of the following criteria: (1) evidence of the acid-fast bacilli (AFB) in histologic sections; (2) positive culture in the endoscopy/colonoscopy sample; and (3) prompt response to antituberculosis treatment. This antituberculosis treatment response was defined as the absence of symptomatology (pain and/or diarrhea) after 6 months of antituberculosis treatment and was assessed by 2 gastroenterologists and one tuberculosis expert from the Peruvian National Program of Tuberculosis (these 3 specialists agreed on the clinical response of the patients). Neither upper endoscopy nor colonoscopy improvement was evaluated. We excluded cases with scarce tissue or insufficient clinical data.

We found a total of 43 patients with the diagnosis of intestinal tuberculosis, but only 33 patients fulfilled the inclusion criteria and thus were recruited for our study.

We collected clinical and endoscopic data of all the patients recruited. The demographic data included: age, sex, diagnosis of lung or multisystemic disseminated tuberculosis, and HIV status. The endoscopic data collected included: the site involved (duodenum, ileum, ileocecal region, right colon, left colon, rectum, or multiple locations), and the type of lesion (erosion, ulcers, polyps, nodular “cobblestone” appearance, or tumor).

The tissue from these patients was subjected to histological examination and ancillary studies (histochemical, immunohistochemical and real-time PCR).

The histologic features were assessed in hematoxylin and eosin (H&E) slides. Each case was re-evaluated by three pathologists separately. The microscopic study evaluated the following histologic features: 1) Granuloma defined as a round or oval collection of epithelioid immune cells in response to a chronic inflammatory stimulus [15]. 2) Confluent granuloma defined as the structures formed by the merge of adjacent granulomas [16]. 3) Microgranuloma: Small nodules composed of 5 to 15 clustered epithelioid histiocytes [17]. 4) Caseation necrosis defined as encapsulated or oval necrotic debris [15]. 5) Giant cells operationalized as present or absent [18]. 6) Crypt architectural disarray defined as elongated, dilated, or branched crypts [19]. 7) Cryptitis is defined as the presence of neutrophils into the surface epithelium and the colonic crypts [20]. 8) Crypt abscess defined as clusters of neutrophils in the crypt lumen [20]. 9) Eosinophils operationalized as the number of eosinophils per high power field in the intestinal lamina propria [21]. 10) Lymphoid follicles or lymphoid aggregated, the first defined as aggregates of lymphocytes with germinal center, and the lymphoid aggregated defined as a collection of lymphocytes and plasma cells without a germinal center [22]. 11) Villus atrophy (evaluated only in the small bowel): It is defined as the decrease in villous height, loss of the normal crypt/villous ratio (3:1), until the complete flattening of the villi [23, 24]. 12) Pyloric metaplasia: Defined as glands in the intestinal mucosa with the characteristics of mucin-secreting distal stomach glands [25].

Paraffin-embedded blocks were stained using the standard protocol of Ziehl–Neelsen to identify an acid-fast bacillus. With this stain, the bacilli stain red, and the background tissue light blue under the effect of methylene blue. A lung tuberculosis sample was used as control tissue.

The immunohistochemistry (IHC) was carried out using the IgG1 type rabbit polyclonal antibody against the BCG antigen of the MT complex (BIO SB inc. Lab, CA, United States). Sections of 3–4 micron were cut from the tissue block and incubated overnight at room temperature. After that, the tissue was deparaffinized and rehydrated. Antigen retrieval solution (DAKO lab) was applied to the tissue, in water bath (98°C) for 30 min, and then cooled for 20 min at room temperature. To remove endogenous peroxidase, methanol and 3% hydrogen peroxide solution were used for 5 min. Primary antibody (anti-*Mycobacterium tuberculosis* rabbit polyclonal antibody in 1:80 dilutions) reacted in a wet environment at room temperature for 60 min and after rinsing the specimens with phosphate buffer saline. A secondary antibody was applied and was left for 30 min to react. Then, the sections were subjected to a polymer-detection complex (DAKO envision) for 30 min. Finally, the Chromogen was applied for 5 min. Positive staining was defined as coarse granular cytoplasmic strong staining.

Real-time PCR is based on the amplification of a fragment of IS6110, which is specific for the *Mycobacterium tuberculosis* complex and found in almost all members of the MT complex. Most MT strains harbor 10–15 copies, located at different chromosomal sites. For real-time PCR sample preparation, the formalin-fixed paraffin-embedded (FFPE) specimens were sliced with disposable sterile blades in each paraffin block and deparaffinized. DNA was extracted from 25–50 mg. tissues using a High Pure PCR Template Preparation kit (Roche Diagnostics, Dresden Germany). The extracted DNA was then used as DNA template for the DiaPlexQ™ MTC/NTM Detection assay (Seegene, Seoul, South Korea). This assay relies on real-time multiplex PCR and distinguishes between MT and non-tuberculosis mycobacteria. Primers for insertion sequence IS6110 for MT detection and the Pan-Mycobacterium 16S rRNA gene for NTM detection were amplified with Bio-Rad CFX96 Touch™ real-time PCR detection system. The real-time PCR mixture was prepared in a total volume of 25 µL, which contained 1–5 µL DNA sample. Real-time PCR was performed under the following conditions: UDG reaction at 50˚C for 3 min, initial PCR activation at 95˚C for 15 min followed by 40 cycles of denaturation at 95˚C for 10 s and annealing at 58˚C for 40 s. Amplifications were performed and results analyzed according to the manufacturer’s instructions.

Descriptive statistics were used to report our findings.

## Results

A retrospective study was carried out at the Hospital Nacional Daniel Alcides Carrion (HNDAC) and Histodiagnóstico Gastrointestinal Private Pathology Center. The 33 patients who fulfilled the inclusion criteria were recruited in the study. 25 patients (75.7%) met the inclusion criteria by the evidence of the AFB in histologic sections, and 8 (24.24%) by the response to antituberculosis treatment, according to the inclusion criteria described in methods. The clinical parameters assessed are shown in Table 1.

**Table 1:** Clinical Data of the 33 patients with intestinal tuberculosis included in the study between the years 2010–2023

Clinical characteristic of the study group	
Age in years (mean ± standard deviation)	41.2 ± 17
Male:Female	23:10
Lung tuberculosis	3
Multisystemic disseminated tuberculosis	3
HIV positive status	4

**Fig. 1:**
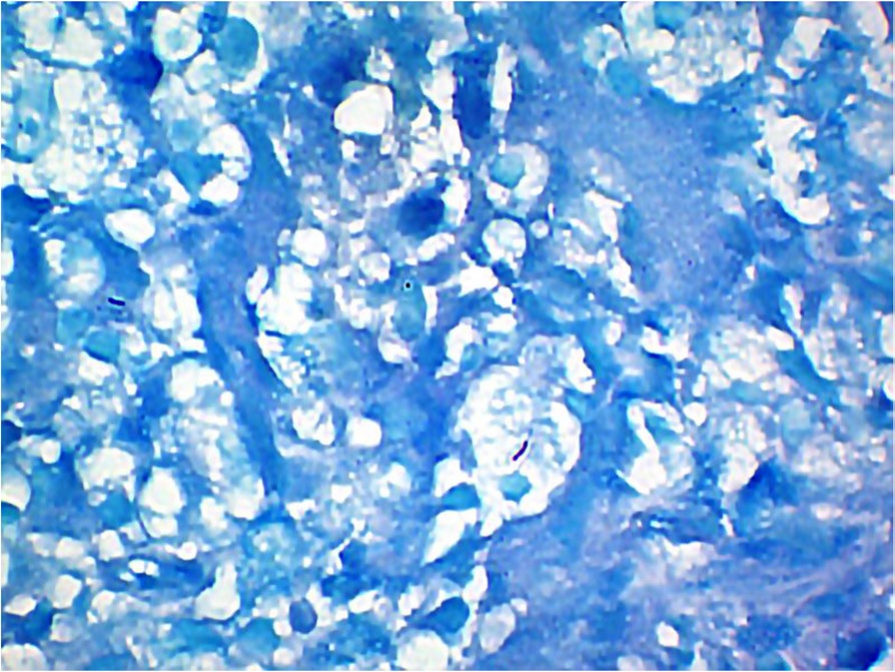
Positive acid-fast bacilli were found with Ziehl–Neelsen stain

Ziehl–Neelsen staining was positive for acid-fast bacilli in most of the patients; nevertheless, most of them showed very few mycobacteria, making the bacilli detection difficult (Fig. 1).

**Fig. 2:**
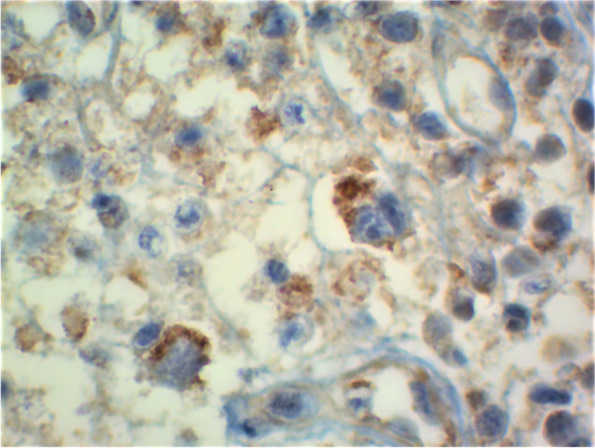
A positive immunohistochemistry was found in the cytoplasm of macrophages

In IHC, the staining was mainly observed in the inflammatory cells, while the necrotic centers were negative for the IHC study (Fig. 2). Occasionally, the surrounding tissue showed weak staining considered as negative background.

IHC was positive in more than 90% of the 33 cases. IHC was also positive in 24 of the 25 (96%) of the Ziehl–Neelsen positive cases, and in 11 of the 12 (91,6%) real-time PCR positive cases. Only 3 IHC negative cases were found, out of which 2 were also real-time PCR negative, and only 1 IHC negative case was real-time PCR and Ziehl–Neelsen positive. Real-time PCR was positive in 38.7% of the cases. All real-time PCR positive cases except one were also positive for acid-fast bacteria Ziehl–Neelsen staining.

Colonoscopy was performed in 28 patients; upper endoscopy, in 4 patients; and both (upper endoscopy and colonoscopy), in 2 patients. The endoscopic findings assessed are shown in Table 2 and the histologic findings in Table 3.

**Table 2:** Intestinal tuberculosis. Endoscopic findings. 2010–2023

**Localization**		
Duodenum	4	12.1%
Ileum	3	9.1%
Ileocecal region	11	33.3%
Right colon	3	9.1%
Left colon	2	6.1%
Rectum	3	9.1%
Multiple locations	7	21.2%
**Type of lesion**		
Erosion	2	6.1%
Ulcers	21	63.6%
Polyp	6	18.2%
Tumor	3	9.1%
Nodular “cobblestone” appearance	1	3%
Total	33 patients	100%

**Table 3:** Intestinal tuberculosis. Histologic findings. 2010–2023

	Number of patients (%)
Granulomas	32 (97%)
Confluent granulomas	27 (81.8%)
Microgranulomas	25 (75.8%)
Caseum necrosis	7 (21.2%)
Giant cells	7 (21.2%)
Crypt architectural disarray	26 (78.8%)
Villous atrophy (only for small bowel)	5 (15.2%)
Pyloric metaplasia	1 (3%)
Cryptitis	19 (57.8%)
Crypt abscess	8 (24.2%)
Eosinophils (mean ± standard deviation)	5.8 ± 7
Lymphoid follicles	20 (60.6%)

The ileocecal region was the most affected area (33.3%) (Table 4); and the cases with multiple involvement of the intestine comprised the ileocecal region as well. The rectal compromise is rare, but it was identified in 3 patients (9%), one of whom was presented with fistulae.

**Table 4:** Intestinal tuberculosis. Ancillary tests. 2010–2023

	Ziehl–Neelsen		IHC		real-time PCR^a^	
	Number	%	Number	%	Number	%
Positive	25	75.8	30	90.9	12	38.7
Negative	8	24.3	3	9.1	19	61.3

^a^In 2 cases the real-time PCR could not be performed because the tissue was consumed during the processing

**Fig. 3:**
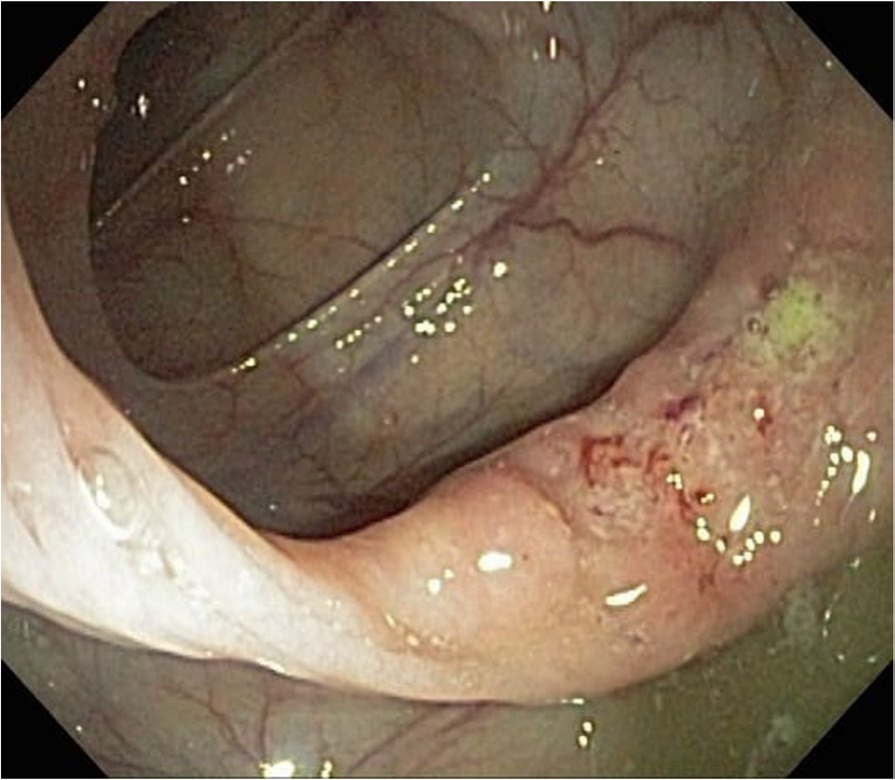
Ulcer lesion at ascending colon

The most frequent endoscopic appearance (in all the intestine segments evaluated) was an ulcer (Fig. 3); and we found two cases with a superficially depressed presentation (Type O-IIc).

**Fig. 4:**
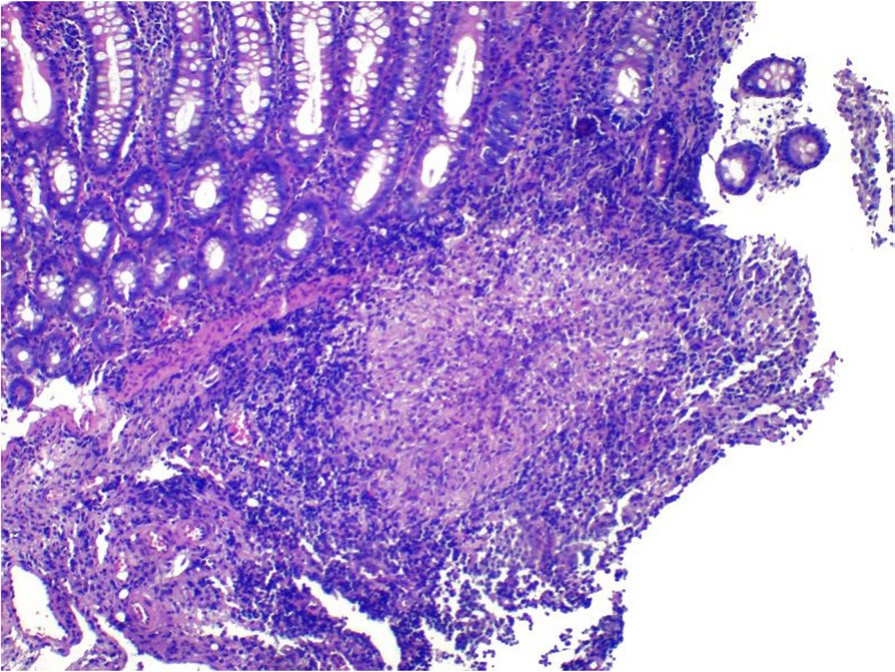
Submucosal granuloma. Mucosa shows mild crypt disarray

**Fig. 5:**
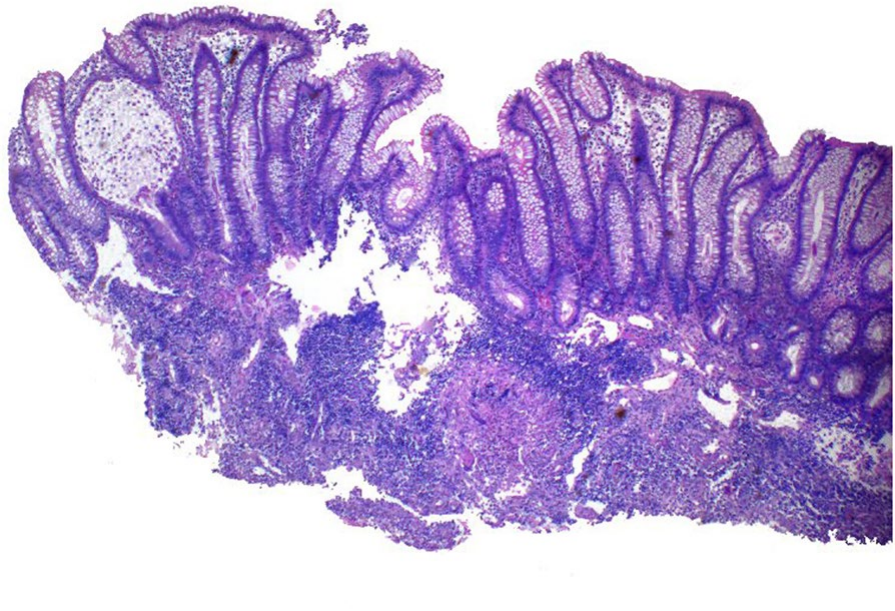
Mild crypt disarray with elongated and dilated crypts. Submucosa granuloma

Histological examination revealed the presence of granulomas in almost all the cases studied. The only case without granulomas was initially misdiagnosed as Crohn's disease. Most of the granulomas were composed solely of epithelioid histiocytes (75.8%). Most granulomas were confluent and found in the submucosa or granulation tissue (Fig. 4). While the glandular architecture of the intestinal mucosa was preserved in 7 patients and most of the cases had mild disarray, a severe crypt disarray with branched crypts was noted in 10 patients (31%) (Fig. 5).

The ancillary tests for the detection of *Mycobacterium tuberculosis* performed on the tissue sample are shown in Table 3.

## Discussion

Our study was focused on determining the positivity rates of auxiliar tissue tests (IHC and real-time PCR) performed for the diagnosis of intestinal tuberculosis, and to describe the endoscopic and histologic findings of these patients. We found a high IHC positivity rate (90.9%); however, the real-time PCR positivity rate was surprisingly low (38.7%). The most affected area was the ileocecal region with the ulcer being the most predominant endoscopic appearance (63.6%). The most frequent histologic finding was the presence of granulomas, which were mostly confluent without giant cells or caseous necrosis, followed by alterations in the architecture of the crypts, which were mild in most cases.

IHC has a better sensitivity than AFB Ziehl–Neelsen staining because it does not require an intact wall cell to be positive, which is very helpful for the diagnosis of paucibacillary tuberculosis [26]; this can explain the high positivity of IHC in our findings (90.9%). However, the specificity of this test is lower, which can result in false-positive reactions due to false-positive background staining. To improve the specificity of IHC some authors recommend proper elimination of background staining, because fine granular staining in the background can cause false-positive reaction [7]. Also, it has been reported that the sensitivity and specificity of IHC could be improved using antibodies against MPT64 antigens rather than antibodies against BCG antigens [27]. MPT64 is a secretory protein of MT, and a specific antigen that differentiates the MT complex from the mycobacteria other than tuberculosis [28]. Currently, the detection of MPT64 protein is performed mainly by immunochromatography. Nevertheless, in immunohistochemistry, the application is limited [29], for this reason, we did not have access to this antibody in our study. To improve the specificity of IHC, the use of monoclonal antibodies instead of polyclonal antibodies has also been recommended [14, 27]. Although, the high price of monoclonal antibodies in Peru made it difficult to access to it. Even so, the IHC employed in our study was the ancillary tissue test with a greater positive percentage in relation to real-time PCR or Ziehl–Neelsen (90.9% versus 38.7% and 75.8%, respectively). A greater percentage of positive cases from the anti-BCG IHC versus the Ziehl–Neelsen stain was previously reported [30].

The immunohistochemistry manifests as a coarse granular staining in the cytoplasm of mainly macrophages and other cells such as neutrophils, lymphocytes, endothelial cells, and dendritic cells [7, 31]. In this study, our immunohistochemistry staining was limited only to macrophages, highlighting the importance of these cells in tuberculosis pathogenesis.

In our study, the real-time PCR positivity rate (38.7%) was lower than that reported in previous studies, which indicated a positivity rate that varies from 60 to 70%, but only in acid-fast bacilli (AFB) positive samples [10, 12]. The low PCR-positivity of our results could be attributed to the small size of the sample [14]; absence or fewer copies of sequence IS6110 in some strains of *Mycobacterium tuberculosis* [10]; or DNA structural changes of paraffin-embedded tissues due to prolonged formalin fixation [12]. These findings raise the possibility that some local mycobacteria might not harbor the sequence IS6110. Better designes studies are required to evaluate this idea.

Ziehl–Neelsen staining for the identification of acid-fast bacilli is the most trustworthy histologic finding in tuberculosis. It should be noted that most of our cases (75%) were positive, which is higher than the findings of previous reports, but this is related to the fact that Ziehl–Neelsen positivity was one of the inclusion criteria of our study. The sensitivity of Ziehl–Neelsen staining for the detection of MT in histological specimens is variable, ranging from 0 to 60% [2, 7, 32, 33]. The difficulty to detect mycobacterium can be explained by its paucibacillary nature [5], the CDC points out that “there must be between 5000–10000 bacilli per milliliter of specimen to allow the detection of bacteria in stained smears” [34], and for each slide at least 10 [4] bacilli [7, 30]. For that reason, it is recommended that at least 8 biopsies should be taken to ensure an optimal microscopic evaluation [6].

In our study, the ileocecal region was the most frequent site affected by tuberculosis which is consistent with previous studies. In fact, TB can compromise any level of the gastrointestinal tract, from the esophagus to the anus [35]; however, the ileocecal region is the most affected area, accounting for 44%-84% of all gastrointestinal tuberculosis cases. [35–37]^.^ Most authors believe that in these areas, the presence of lymphoid tissue and the physiologic stasis of the cecum promotes mycobacterial growth. [38] This predilection for ileocecal region complicates the differentiation of tuberculosis from Crohn’s disease, where ileocecal involvement is similar [39].

While upper gastrointestinal tract involvement by tuberculosis is rare [35], we found a greater rate (12.1%) of duodenum involvement than previously reported (1%-6%) [35, 40–42]. Multisegmented involvement of the colon has been rarely reported [26, 35]. Only one report indicates the colonic involvement distribution in single and multiple site lesions (31% and 36%, respectively) [36]. In our study, multisegmented colonic involvement was found in 21.2% of cases.

In our study, ulcer was the most frequent endoscopic finding, mostly reported by the gastroenterologists as multiple, consistent with previous reports [14, 36, 43]. However, the endoscopic presentation of intestinal tuberculosis is quite variable and also includes tumors, strictures, erosions, and nodularity of the mucosa [36, 43, 44]. These endoscopic findings, however, were present only in a small group of patients in our study. The distinction between intestinal TB from Crohn´s disease ulcer can be a true challenge, especially when nodularity of the mucosa layer is described as “cobblestone” [36, 45–48]. This endoscopic finding has been regularly related to Crohn´s disease, which makes the differential diagnosis even more difficult; however, four parameters have been proposed. These parameters would be more common in Crohn's disease than in intestinal TB, and they are: aphthous ulcers, longitudinal ulcers, anorectal lesions, and cobblestone appearance [49]. Additionally, the mucosa surrounding intestinal TB ulcers tends to be abnormal (edematous, erythematous, irregular, or nodular), whereas the mucosa surrounding Crohn´s disease ulcers tends to be normal [36].

Stricture and tumor appearance are the least frequent endoscopic findings [2], but they are the most concerning due to their similarity with colon neoplasia. We found only 4 cases described as a “tumor” in the colonoscopy report. In this scenario, a meticulous histological study of the sample will be essential for diagnosis as reported in previous papers [50–53].

Histological evaluation of intestinal tuberculosis poses a diagnostic challenge for pathologists, since it must be differentiated from Crohn´s disease. Both diseases can exhibit granulomatous reaction. Crohn's disease granulomas are composed mainly of clusters of epithelioid cells without giant cells or necrosis [54, 55]. Conversely, tuberculosis granulomas are usually reported as large (> 400 µm), confluents, and contain giant cells called “Langhans giant cells” [56], along with caseous necrosis [48, 57, 58]. Interestingly, a study reported that only between 13 to 33% of the patients with intestinal tuberculosis showed those findings [5]. Our group of patients with intestinal tuberculosis presented mostly incomplete granulomas; only 7 (21.2%) cases had complete granulomas with caseum necrosis. This finding differs from other publications that found caseating granulomas in 70.6% and 40% respectively [2, 48]. We suggest considering non-caseating granulomas as a common histologic finding in intestinal tuberculosis in our population.

Other histologic features such as ulcers lined by bands of epithelioid histiocytes, disproportionate submucosal inflammation, and submucosal granulomas are more frequent in intestinal tuberculosis than in Crohn’s disease [45]. Moreover, in intestinal tuberculosis, ulcers usually do not penetrate beyond the muscularis, and granulomas are commonly located under the ulcer bed [9]. Most of our cases presented granulomas located in the submucosa rather than in the mucosa, thus it is recommendable that deeper biopsy should be taken from the margins of the ulcers to reach the submucosa [4].

Crypts disarray, presence of lymph follicles, and eosinophils counts were other histologic features assessed in our study. We found a predominantly mild crypt disarray and a very few crypts branching. Intestinal tuberculosis has a chronic course, which explains the alteration of the normal architecture; however, these changes are not as prominent as in inflammatory bowel disease [35]. Lymph follicles could play a role in tuberculosis pathogenesis since they can harbor granulomas. Nearly all patients in our study had lymph follicles in the mucosa, and rarely in the submucosal layer.

Although tuberculosis cases at the national and global level continue to increase [59, 60],

its diagnosis remains a challenge, particularly in cases with intestinal involvement, where histologic findings could be similar to other diseases like colon carcinoma or Crohn’s disease [36, 39, 45]. Additionally, microbiologic culture, the gold standard in the diagnosis of *Mycobacterium tuberculosis* [5, 6]; takes several weeks to be obtained, and the paucibacillary nature of these microorganism could lead to report a false negative [61]. This highlights the importance of using other ancillary tests for the diagnosis on intestinal tuberculosis. In that regard, our study is the first retrospective study conducted in Latin America to evaluate the positivity rates of IHC and real-time PCR in tissue sample obtained from endoscopy in the diagnosis of intestinal TB. In Peru, these tests are infrequently performed due to their high cost, which can be up to three times higher for IHC or thirteen times higher for real-time PCR compared to a conventional study (histologic study + Ziehl Neelsen).

It should be noted that there are some limitations in the present study: the small sample size, and the retrospective nature (which lacks a systematic prospective follow-up) therefore definitive conclusions from the study cannot be made. Besides, including “prompt response to antituberculosis treatment” as an inclusion criterion could have increased the false positivity rate, although it must be highlighted that most of our cases were diagnosed with Ziehl–Neelsen positive in histology.

In conclusion, despite these limitations, our data shows a high positivity rate of anti-BCG IHC in the diagnosis of intestinal tuberculosis, even in Ziehl–Neelsen negative cases. Thus, it should be considered especially in difficult cases with a Ziehl–Neelsen negative staining to avoid misleading the diagnosis; unlike real-time PCR, which had a low positive rate in our study. Finally, we found that a multicentric analytic study should be performed to establish the sensitivity and specificity of the histologic tests used in our study (anti-BCG IHC, real-time PCR and Ziehl–Neelsen stain), and other ancillary tests not evaluated in this study such as anti-MPT64 IHC.

### Supplementary Information

**Supplementary Material 1. **

## Data Availability

The data can be provided by the corresponding author on reasonable request.
